# Theory of Mind and Sociometric Peer Status: The Mediating Role of Social Conduct

**DOI:** 10.3389/fpsyg.2018.02191

**Published:** 2018-11-12

**Authors:** Antonia Lonigro, Roberto Baiocco, Susanna Pallini, Fiorenzo Laghi

**Affiliations:** ^1^Department of Social and Developmental Psychology, Università degli Studi di Roma La Sapienza, Rome, Italy; ^2^Department of Education, Università degli Studi Roma Tre, Rome, Italy

**Keywords:** theory of mind, sociometric peer status, prosocial behavior, internalizing behavior, externalizing behavior

## Abstract

The present study aimed at investigating the mediating role of social conduct in the relation between theory of mind (ToM) and sociometric peer status. One hundred and seventy-seven 8- to 11-year-olds filled out a battery encompassing advanced ToM skill, verbal ability and sociometric peer status, expressed in terms of social preference and social impact. A questionnaire on students’ externalizing, internalizing and prosocial behaviors was administered to teachers. Only externalizing behavior mediated the link between ToM and social impact, controlling for age, gender, and verbal ability. Implications and suggestions for future research were discussed.

## Introduction

Theory of mind (ToM) is the common expression to denote the specific human ability to infer and differentiate own one’s and others’ mental states. This knowledge allows people to predict and explain human behavior and, overall, to understand the link between the content of mind and action (Flavell, 2004). Although the precursors are identified at very young age (Bellagamba et al., 2012; Yott and Poulin-Dubois, 2016), the official starting point for ToM acquisition is between the ages 3 and 5 years (Camaioni, 1995). Most 4-year-olds successfully pass the first-order false-belief tasks, revealing that they are able to contemporary hold in their own mind the knowledge of the reality and realize that a person may have a false belief (Walker and Murachver, 2012). Over elementary school years, the second developmental step is attained: Second-order reasoning. By 9–10 years of age, most children infer the content of mind of a person about the content of mind of another person (Miller, 2009).

How the development of more advanced mind reading abilities from preschool to school years may affect children’s social life and encourage effective forms of interaction with classmates has been the most popular topic among studies in the last three decades (for meta-analysis, see Slaughter et al., 2015). In them, the sociometric approach, which concerns peer–based assessments of the extent to which children are liked or disliked by their classmates, has been commonly used to draw a complex picture of social interactions at school and determine peer status. Specifically, this approach, as proposed by Peery (1979) and revised later (Coie et al., 1982; Newcomb and Bukowski, 1983), frames interpersonal functioning of each pupils in terms of social impact and social preference. Both of them derive from positive (like most) and negative (like least) nominations expressed for each child by her/his own classmates. However, social preference is obtained by subtracting liked-least nominations from liked-most nominations, thus indicating the child likability. Children receiving more positive nominations and lower negative nominations obtain a high social preference index and are classified as popular children. Conversely, children with a low social preference index, whereby negative nominations outperform positive nominations, are labeled as rejected by their peers.

Social impact is derived by summing both types of nominations and mirrors the degree to which a child is noticed or visible in the peer group (Bellmore et al., 2010). In case of high social impact, it means that children are both liked and disliked by classmates. They are commonly defined as controversial children. When both the positive and negative nominations are few, low social impact is obtained. This is the case of children who are neglected and not noticed by their classmates (Bellmore et al., 2010).

Previous studies have revealed mixed findings on the relation between mental states understanding and peer status. In the majority of them involving both preschoolers and school-aged children, ToM skills are positively and significantly linked to popularity among peers (Banerjee and Watling, 2005; Braza et al., 2009; Banerjee et al., 2011; Caputi et al., 2012; Fink et al., 2014, 2015). In other studies, no significant and concurrent correlations were reported (Slaughter et al., 2002; Diesendruck and Ben-Eliyahu, 2006), even controlling for age and verbal abilities (Watson et al., 1999; Flynn and Whiten, 2012). The recent meta-analysis carried out by Slaughter et al. (2015) has shed light on this topic, analyzing 20 different studies with children aged from 3 to 10 years. The authors have revealed that children’s ability to understand others’ mental states is positively tied to their concurrent peer popularity. This association is weaker among boys than girls, whereas no age difference is found. The mean effect sizes for both preschool-aged and school-aged cohorts are identical (rs = 0.18) and statistically significant. However, it must be said that children’s correct performance on ToM tasks accounts for about 4% of the variance in peer popularity. How hypothesized by the authors of the meta-analysis, this small effect may be due to the presence of other factors - cognitive, personality, physical and behavioral – that affect children’s sociometric status. Badenes et al. (2000) have previously realized how the impact of ToM abilities on peer status may be influenced by prosocial motivation. Furthermore, in this meta-analysis the role of verbal ability had not been considered, even if in the literature it is a well-established correlate of ToM (for meta-analysis, see Milligan et al., 2007). The present study aimed at overcoming the limits described in the meta-analysis, testing the impact of social conduct on the relation between ToM and social preference and social impact, controlling for language ability, gender and age.

Social conduct is a general term to indicate the array of behaviors engaged by children during social interactions. In this study, we focused on 3 clusters of behaviors – prosocial, internalizing and externalizing – that have appeared to be associated with indices derived by positive and negative nominations (LaFontana and Cillessen, 2002; Lease et al., 2002; Keane and Calkins, 2004; Horner et al., 2010). Prosocial behavior is defined by Eisenberg et al. (2006) as *“voluntary and intentional behavior intended to benefit other,”* encompassing helping, sharing, and caring. Internalizing behavior and externalizing behavior are conceptualized as 2 broad categories of problematic conduct, whose key feature is the difficulties in emotion regulation. If the tendency to withdraw and take inside distress is the hallmark of internalizing behavior (e.g., anxiety, depression, shyness, somatic symptoms), the tendency to direct and act out distress characterizes externalizing behavior (e.g., aggressiveness, rule breaking, delinquent act) (Hatoum et al., 2018).

Past research has largely documented how social conduct is predictive of the degree to which a child is liked or disliked by classmates at different ages (LaFontana and Cillessen, 2002; Lease et al., 2002; Keane and Calkins, 2004). Pupils with a higher social preference engage in more prosocial behavior, appear more sociable and less aggressive, and obtain better academic performance than pupils with lower social preference. A more recent study (Horner et al., 2010) has confirmed past findings, using peer-reported problem (e.g., overt and relational aggression, and impulsiveness) and prosocial behavior evaluations among elementary school children. The authors found that higher liked-least nominations are often more positively associated with problematic behavior and negatively with prosocial behavior compared to higher liked-most nominations. Popular status (higher liked-most nominations than liked-least nominations) is positively related to prosocial behavior and negatively with all indexes of problematic conduct. Controversial category (both high liked-most and liked-least nominations) is positively related to both problem behavior and prosociality. The opposite pattern is found among neglected children (both low liked-most and liked-least nominations).

Based on the aforementioned investigations on peer status and social behavior (LaFontana and Cillessen, 2002; Lease et al., 2002; Keane and Calkins, 2004; Horner et al., 2010), and prior empirical findings from the ToM literature (Badenes et al., 2000; Fink et al., 2014, 2015, Slaughter et al., 2015), we hypothesized a model in which ToM ability would positively impact on prosocial behavior and social preference and negatively on social conduct, distinguishing prosocial, internalizing and externalizing behavior. Furthermore, the mediating role of social conduct in the relation between ToM and social impact and social preference was tested.

## Materials and Methods

### Participants

One hundred and seventy–seven children constituted the final sample of the study (87 girls; age range = 8–11 year; average age = 9 year and 7 month; *SD* = 6 months). They attended the fourth and fifth classes (total class = 10; class size ranged from 18 to 23 pupils) of the three elementary schools in the middle–class districts of a large city in the center of Italy. All pupils which were enrolled in the study spoke Italian as their first language and none of them had deficit in cognition, language, and learning nor were receiving special education or speech/language services. All this information has been provided by the teachers. The did not directly test the children’s abilities, but in case of a diagnosis this was established by a medical/psychological task force and, then, shared with the teachers to plan a specific intervention at school.

### Procedure

Children were recruited by sending an information sheet on the research project and consent form to the principals of the elementary schools and parents of all enrolled pupils. Obtained the voluntary consensus, two individual sessions occurred during which the pupils’ abilities were tested individually by a female research assistant trained by the first author. As a whole, two sessions lasted about 50 min and placed in a quiet school area provided by the principals. For each child the two testing occasions took place later than 2 weeks from each other.

Theory of mind abilities were evaluated in Session 1 through the use of brief stories. Each story and its related questions were presented on a sheet, which was offered the child with the request to follow when the research assistant read out the story aloud. Only five children required to read the stories themselves. Session 2 was devoted to assessing the children’s verbal ability and administering the peer nomination task.

The teachers who spent with pupils at least the last 2 years were invited to fill out a questionnaire investigating prosocial, internalizing, and externalizing behaviors. A total of 10 teachers were involved, one for each classroom. Thus, each pupil has been assessed once by one teacher.

### Measures

#### Theory of Mind

The version of stories provided by Gini (2006) has been adopted to investigate the children’s ability to infer inner states. The stories are not completely original, but they constitute a translation of the Strange stories developed by Happé (1994) and those used by Sutton et al. (1999). A total of 10 stories were administered. Half of them are called cognitive stories because they test the ability to infer epistemic mental states (beliefs, intentions, and thoughts) held by the story protagonists. The remaining half stories are labeled emotional and they involve the understanding of non-epistemic mental states. Specifically, emotional stories tap the ability to distinguish the emotions really felt from those expressed by the story protagonist to other characters. Only for emotional stories, pictures of faces expressing different emotions (e.g., happiness, sadness, anger, and a neutral face) were presented to the participants in order to help them with their answers.

Each story is followed by a control question, whose aim is to verify if the child has understood the real state of events told in the stories, and 2 experimental questions assessing the understanding of mental states or emotions (for details, see the Appendix). Control questions are asked before the experimental questions. If the control questions are answered incorrectly, ToM abilities are not evaluated. For experimental questions, children score 0 if they do not answer the question, 1 if their answer is not correct, 2 if they answer right but without referring mental state, and 3 if they give a complete answer encompassing the reference to inner state. As claimed by Gini (2006) in his study, the stories appear suitable and valid for primary school pupils.

Two independent raters coded all the answers (Cohen’s Kappa for 20% of the children was 0.93). When controversial evaluation occurred, a third independent rater helped to solve the doubts. The total score was obtained by summing the story scores (range from 0 to 30).

#### Social Behavior

The Italian version of the Strength and Difficulties Questionnaire (SDQ – ITA; Goodman et al., 1998) consists of 25 items, which are divided into 5 subscales, each constitutes by 5 items. The questionnaire covers emotional problems, conduct problems, hyperactivity problems, peer problems and prosocial behavior. Each item is evaluated across three-point Likert format (0 = not true, 1 = somewhat true and 2 = certainly true). As proposed by Goodman et al. (2010) for analysis in low–risk samples, the broader internalizing and externalizing SDQ scales were adopted. The sum of emotional and peer subscales constitutes the Internalizing scale (range 0–20), whilst hyperactivity and behavioral subscales allow to obtain the Externalizing scale (range 0–20). The children’s tendency to act prosocially is evaluated through the Prosocial scale. The SDQ – ITA was administrated to teachers. A reliability analysis on the three subscales indicated acceptable levels of internal consistency (Cronbach’s α = 0.75 for internalizing behavior; Cronbach’s α = 0.80 for externalizing behavior; Cronbach’s α = 0.86 for prosocial behavior).

#### Receptive Language

The Italian version of the Peabody Picture Vocabulary Test – Revised (PPVT – R, Stella et al., 2000) is designed the children’s ability to understand spoken words in Italian. Raw scores were used in all analyses.

#### Measurement of Peer Status

Children’s peer status was determined following the methodological recommendation by Coie and Dodge (1983) and adopting the same procedures as in Slaughter et al. (2002). Specifically, children were required to nominate the three classmates they liked (like most nominations) and the three classmates they dislike (like least nominations) spending the recess time, respectively. Once standardized both liked-most and liked-least nomination scores for each child within each classroom group, these indices allow to compute a social preference (SP) score and a social impact (SI) score, which in turn need to be standardized. SP is defined by liked-most nomination minus liked-least nominations, whereas SI is determined by summing both the kinds of nominations.

### Statistical Analyses

Preliminary analysis was carried out using SPSS 19.00 for Windows. Bivariate correlations among key variables of the study were computed. The robust maximum-likelihood estimation method in LISREL 8.7 with the SIMPLIS syntax (Jöreskog and Sörbom, 1993) was used to test our hypotheses. The goodness of the hypothesized model was determined by the values of both absolute and incremental fit indices. Specifically, chi squared test, which needs to be non-significant, and the root mean square error of approximation (RMSEA), whose value would be equal to 0.06 or less, constitute the most widely known absolute indices and were adopted in the current study. Among incremental fit indices, the comparative fit index (CFI) and the non-normed fit index (NNFI) were considered. Models with the CFI equal or superior to 0.95 and NNFI equal or superior to 0.90 are considered acceptable (for details on cut-off criteria for fit indexes, see Hu and Bentler, 1999).

## Results

### Preliminary Analysis

Table 1 provides means and standard deviations for each story, the dimensions derived from the SDQ, and the receptive language considered the full sample, whereas correlations among the variables measured in the study were inserted in Table 2. ToM was positively and significantly related with age, verbal ability, prosociality and social preference, and negatively with both internalizing and externalizing behaviors. Positive and highly significant correlations were found between internalizing and externalizing behaviors, and between prosociality and verbal ability. Gender was positively associated with prosociality and negatively with language and externalizing behavior. Social preference was negatively and significantly related with internalizing and externalizing behaviors. A negative correlation was found between prosociality and problem behaviors (both internalizing and externalizing). Finally, social impact correlated with externalizing behavior positively and in a significant way.

**Table 1:** Means and standard deviations for social conduct, receptive language, and stories considering their underpinning conceptual knowledge.

Story type	Means	Standard deviations
Belief (Story 1)	1.99	0.58
Belief (Story 2)	2.03	0.56
Double bluff (Story 3)	1.99	0.98
Persuasion (Story 4)	2.28	0.75
Misunderstanding (Story 5)	2.48	0.82
White lie (Story 6)	2.03	0.86
Deceit (Story 7)	2.34	0.73
Deceit (Story 8)	2.24	0.70
Misunderstanding (Story 9)	1.77	0.72
Belief (Story 10)	1.77	0.67
total score	20.88	3.53
**Social conduct**		
Prosocial behavior	8.72	1.94
Externalizing behavior	1.76	2.99
Internalizing behavior	1.68	2.51
**Language**		
Receptive language	134.62	17.46


**Table 2:** Pearson’s product moment correlations for the variables measured in the study (*N* = 177), gender (dummy variable, with girls scoring higher than boys) and age.

Study variables	1	2	3	4	5	6	7	8	9
(1) Gender	–								
(2) Age	0.07	–							
(3) Receptive language	–0.25**	0.13	–						
(4) Theory of mind	0.12	0.24**	0.37***	–					
(5) Internalizing behavior	–0.09	0.07	–0.16*	–0.36***	–				
(6) Externalizingbehavior	–0.24**	–0.06	–0.04	–0.23**	0.46***	–			
(7) Prosocial behavior	0.25**	0.42***	0.04	0.27***	–0.22**	–0.43***	–		
(8) Social preference	0.12	0.11	0.10	0.24**	–0.24**	–0.31***	0.11	–	
(9) Social impact	–0.06	0.05	0.06	0.11	0.03	0.29***	–0.05	0.01	–


*^∗^p < 0.05, two-tailed. ^∗∗^*p* < 0.01, two-tailed. ^∗∗∗^*p* < 0.001, two-tailed.*

### ToM, Social Conduct and Sociometric Status: Direct Effects

**FIGURE 1:**
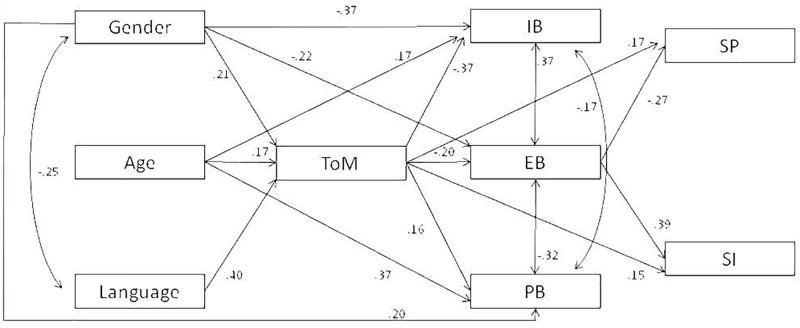
Direct effect path model. ToM, theory of mind; IB, internalizing behavior; EB, externalizing behavior; PB, prosocial behavior; SP, social preference; SI, social impact. Non-significant regression paths are not shown.

Paths were specified from ToM to prosocial behavior, social preference and social impact (positive associations expected), and to internalizing and externalizing behaviors (negative associations expected). Furthermore, prosocial and problem behaviors were regressed on social preference and social impact. The effects of age, gender and verbal ability were also controlled for all variables inserted in the hypothesized model. The model had a good fit, MLR: χ^2^(6) = 2.72, *p* = 0.84, RMSEA = 0.000, CFI = 1.00, NNFI = 1.08. As it is possible to see in Figure 1, all control variables were positively associated with ToM. Prosocial behavior was negatively predicted by internalizing and externalizing behaviors, and positively by gender, age, and ToM. Internalizing behavior was positively associated with externalizing behavior and age, and negatively with ToM. Negative paths from externalizing behavior were found with prosocial behavior, gender and ToM. Social preference was positively associated with ToM and negatively with externalizing behavior. Finally, positive paths were obtained from social impact and ToM and externalizing behavior.

### ToM and Social Status: The Mediating Role of Social Conduct

In order to proceed in exploring the mediating model, three conditions were required: (1) the direct effect of the predictor (ToM) on the mediators (prosocial, internalizing and externalizing behaviors, respectively), and (2) on the outcome variables (social preference and social impact, respectively), and (3) the direct effect of the mediator on the outcome variables (for details, see Baron and Kenny, 1986; Kenny and Judd, 2014). The three conditions were fulfilled only for externalizing behavior. Thus, we tested the mediating role of externalizing behavior on the relation between ToM and social preference and social impact, controlling for age, gender, and verbal ability. To do this, Sobel test was run (MacKinnon et al., 1995), yielding a significant effect of externalizing behavior in mediating the association between ToM and social impact (*z* = -2. 16, *p* < 0.05). The mediated effect accounted for 28%of the total effect of ToM on social impact.

Results also showed that the indirect effect of ToM on social preference through externalizing behavior was very close to statistical significance (*z* = 1.94, *p* = 0.052).

## Discussion

The present study was designed to verify the mediating role of social conduct, as described by prosocial, internalizing and externalizing behaviors, in the relation between ToM and sociometric status in terms of social preference and social impact. The results show that children’s mind reading ability is a significant predictor of both social preference and social impact, controlling for age, receptive language and gender. This finding adds to a wide array of studies (Hughes and Leekam, 2004; Slaughter et al., 2015; Imuta et al., 2016; Longobardi et al., 2016), highlighting how ToM significantly influences children’s everyday social experiences and interactions.

When the mediating role of social conduct was analyzed, only externalizing behavior met the criteria. ToM was negatively associated with social impact through low scores on externalizing behavior scale. This issue appears to be consistent with neglected status and what it is known about them in the literature (Nelson et al., 2009). Overall, neglected children are not actively dislike by their classmates as it happens for rejected children, who exhibit aggressive and disruptive behaviors (Cantrell and Prinz, 1985). Conversely, neglected children are just not noticed. Their conduct at school is marked by low levels of both sociability and aggressiveness (Newcomb et al., 1993), thus leading neglected children to be not noticed into the classroom. In some research, social withdrawn shown by neglected children is accounted by their shyness or by their poor interest toward making friends (Gifford-Smith and Brownell, 2003; Sette et al., 2017). Our study appears to suggest that neglected children hold the mind reading abilities who should allow them to have social relations. However, how it is well documented in the literature, ToM skills are necessary but not sufficient to social life (Astington, 2003; Lonigro et al., 2014). Hence, which other socio-cognitive and personality variables may affect social behavior among neglected children need to be explored in further research.

Our study also found that the mediation is approaching significance between ToM and social preference through externalizing behavior. Although further investigation is required, this issue appears in line with what it is well established in the literature on popular status and ToM skills (Gifford-Smith and Brownell, 2003; Banerjee et al., 2011). As a whole, popular children often demonstrate sophisticated socio-cognitive abilities and higher levels of cooperativeness and supportiveness than all other classmates classified as rejected, controversial and neglected. For these reasons, they are very appreciated by other pupils.

To sum up, lower scores on externalizing behavior scale appear to be determinant, in association with good performances on ToM stories, on higher likability and lower visibility. However, we believe that other variables underpinning socio-cognitive abilities (e.g., affective empathy) and personality characteristics (e.g., openness to experiences or sociability) may influence the number of liked-most and liked-least nominations. These possibilities may be explored in future research. Moreover, unlike our hypotheses, both internalizing and prosocial behaviors did not affect the relation between ToM and sociometric peer status. In assessing social conduct, a teacher report was adopted, while sociometric status was derived from students’ nominations. Although teachers spent a lot of time with their students, they may not have an exhaustive access to the dynamics of social interactions (Rodkin and Hodges, 2003; Newman and Murray, 2005). Perhaps, different results on the mediating role of social conduct might be obtained if student or parent reports are used.

Finally, we did not test alternative models. Future research with longitudinal designs may ascertain the directions of paths between sociometric status, socio-cognitive abilities and social behavior.

## Ethics Statement

The study was approved by the Sapienza, University of Rome, Research Ethics Board, Department of Social and Developmental Psychology. Written informed consent was obtained from the parents/legal guardians of all participants.

## Author Contributions

AL and FL designed the study, analyzed the data, and wrote part of the results. AL, SP, RB, and FL executed the study, collaborated with the design and writing of the study, and collaborated in the writing and editing of the final manuscript.

## Conflict of Interest Statement

The authors declare that the research was conducted in the absence of any commercial or financial relationships that could be construed as a potential conflict of interest.
