# Supplementary material for: Theory of Mind and Sociometric Peer Status: The Mediating Role of Social Conduct
Source: Front Psychol. 2018 Nov 12;9:2191. doi: 10.3389/fpsyg.2018.02191 (PMC6240647; doi:10.3389/fpsyg.2018.02191)
Supplement: Supplementary file 1 [file Data_Sheet_1.pdf]

Appendix

ToM task

Cognitive story

During the war, the Red army captured a member of the Blue army. They want him to tell them where his army's tanks are; they know they are either by the sea or in the mountains. They know that the prisoner will not want to tell them, he will want to save his army, and so he will certainly lie to them. The prisoner is very brave and very clever, he will not let them find his tanks. The tanks are really in the mountains. Now when the other side asks him where his tanks are, he says, "They are in the mountains."

Control question

Is it true what the prisoner said?

Experimental questions

Where will the other army look for his tanks?

Why did the prisoner say what he said?

Emotional story

Gianni wants to go out with his friends, but he has a really bad tummy ache. He knows that if his mum notices he is ill, she won't let him go out to play. Gianni goes downstairs and asks hi mum: "Can I go out to play?".

Control question

Which picture shows how Gianni really feels?

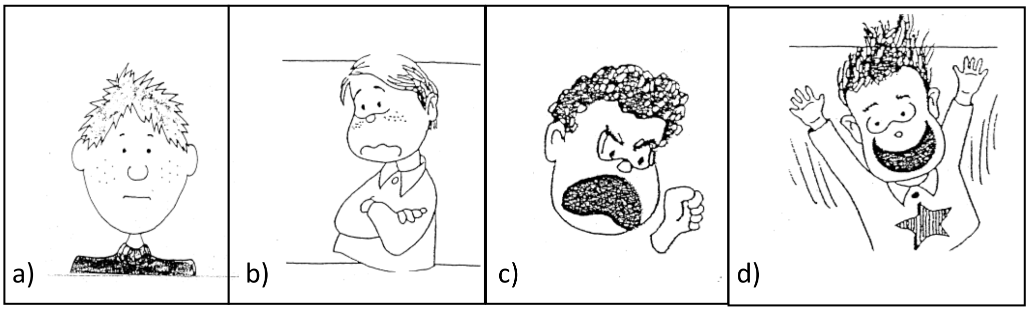

Experimental questions

Which picture shows how Gianni will look like when he talks his mum?

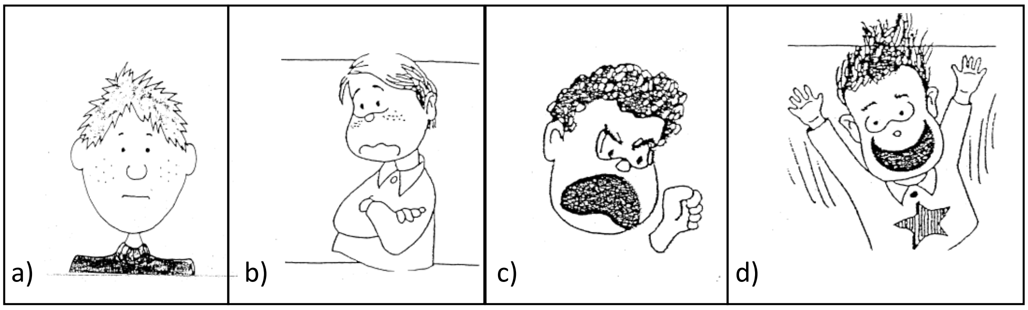

Why?

Peer Status

Who do you like spending recess with? Please, indicate 3 classmates.

Who do you dislike spending recess with? Please, indicate 3 classmates.
